# Effect of low-pressure pneumoperitoneum on pain and inflammation in laparoscopic cholecystectomy: a randomized controlled clinical trial

**DOI:** 10.1186/s13104-023-06492-y

**Published:** 2023-09-28

**Authors:** Mohammad Rashdan, Salam Daradkeh, Mutasim Al-Ghazawi, Jareer Heider Abuhmeidan, Azmi Mahafthah, Ghada Odeh, Mohammad Al-Qaisi, Ikram Salameh, Shahed Halaseh, Lana Al-Sabe, Yousef B. Ahmad, Tuqa Al-Ghazawi, Mahmoud Al-Said, Shereen Sha’bin, Hanan Mansour

**Affiliations:** 1 Department of General Surgery/ Minimally invasive surgery, School of Medicine, Jordan University Hospital, The University of Jordan, Amman, Jordan; 2 Department of General Surgery/Hepatobiliary Division Jordan University Hospital, School of Medicine, The University of Jordan, Amman, Jordan; 3 Department of Biopharmacutics and Clinical Pharmacy, The University of Jordan, Amman, Jordan; 4 Dr. Mohammed Alfagih Hospital, Riyadh, Saudi Arabia; 5 Department of Microbiology, School of Medicine, Jordan University Hospital, The University of Jordan, Amman, Jordan; 6 Department of General Surgery, School of Medicine, Jordan University Hospital, The University of Jordan, Amman, Jordan; 7 King Hussein Cancer Center, Amman, Jordan; 8 Department of General Surgery, School of Medicine, Jordan University Hospital, The University of Jordan, Amman, Jordan; 9 Department of Emergancy Medicine, School of Medicine, Jordan University Hospital, The University of Jordan, Amman, Jordan

**Keywords:** Clinical trial, Low pressure insufflation, Pneumoperitoneum, Laparoscopic cholecystectomy, Pain, Inflammatory marker

## Abstract

**Objective:**

We aim to assess the effect of low-pressure pneumoperitoneum on post operative pain and ten of the known inflammatory markers.

**Background:**

The standard of care pneumoperitoneum set pressure in laparoscopic cholecystectomy is set to 12–14 mmHg, but many societies advocate to operate at the lowest pressure allowing adequate exposure of the operative field. Many trials have described the benefits of operating at a low-pressure pneumoperitoneum in terms of lower post operative pain, and better hemodynamic stability. But only few describe the effects on inflammatory markers and cytokines.

**Methods:**

A prospective, double-blinded, randomised, controlled clinical trial, including patients who underwent elective laparoscopic cholecystectomy. Patients randomised into low-pressure (8–10 mmHg) vs. standard-pressure (12–14 mmHg) with an allocation ratio of 1:1. Perioperative variables were collected and analysed.

**Results:**

one hundred patients were allocated, 50 patients in each study arm. Low-pressure patients reported lower median pain score 6-hour post operatively (5 vs. 6, *p*-value = 0.021) in comparison with standard-pressure group. Eight out of 10 inflammatory markers demonstrated better results in low-pressure group in comparison with standard-pressure, but the effect was not statistically significant. Total operative time and surgery difficulty was not significantly different between the two groups even in the hands of inexperienced surgeons.

**Conclusion:**

low-pressure laparoscopic cholecystectomy is associated with less post operative pain and lower rise of inflammatory markers. It is feasible with comparable complications to the standard of care. Registered on ClinicalTrials.gov (NCT05530564/ September 7th, 2022).

## Introduction

Laparoscopic cholecystectomy was first performed by professor Med Erich Mühe of Böblingen, Germany, on September 12, 1985 [1]. It became the gold standard procedure to remove the gall bladder for different indications [2]. Majority of laparoscopic procedures require the creation of pneumoperitoneum to establish a working space, the standard pressure used is set at 12–15 mmHg [3]. Low pressure pneumoperitoneum 4] has been challenging the complications and feasibility of standard pressure pneumoperitoneum [5, 6]. Clinical trials have compared low pressure vs. standard in terms of perioperative complications, post-operative pain, hospital length of stay, stress response and surgeon comfort [6, 7]. In this trail we conducted a randomized comparison between low pressure and standard pressure pneumoperitoneum for laparoscopic cholecystectomy in terms of postoperative pain, inflammatory markers, timing of surgery and surgeon comfort. This trial adds to the literature the extensive study of inflammatory markers associated with stress of surgery, and the surgeon comfort level along with safety of the procedure with performed by non-experienced surgeons (senior residents).

## Methodology

The study has been reported in line with the CONSORT statement [8]. It is registered on ClinicalTrials.gov under the identifier NCT05530564 and approved by the hospital institutional review board (IRB).

### Design and participants

This is a clinical trial, designed to look for a statistical difference in pain and inflammatory markers after operation, comparing two parallel groups, with an allocation ratio of 1:1. Participants were eligible for the study if they were above the age of 12 years old, with low anaesthesia risk defined as American Society of Anaesthesiologists (ASA) score 1 or 2, and having symptomatic gallbladder stone disease whom were booked for elective laparoscopic cholecystectomy. The study is prospective, all patients admitted during the study period were assessed for eligibility, the study stopped enrolment when hundred eligible patients were selected. The enrolment period extended over a year, from January 2020 to January 2021. Patients not included in the study were those currently or previously diagnosed with acute cholecystitis confirmed by ultrasonography, or those who underwent previous upper gastrointestinal surgeries except bariatric and anti-reflux surgeries, or those currently on immunosuppressants, or currently diagnosed with drug addiction, also pregnant and breastfeeding females were excluded.

### Intervention

Those in the intervention group were scheduled to undergo laparoscopic cholecystectomy at a low-pressure pneumoperitoneum defined as gas insufflation set point between 8 and 10 mmHg, and those in the control group were scheduled to undergo laparoscopic cholecystectomy at a standard-pressure pneumoperitoneum defined as gas insufflation set point between 12 and 14 mmHg. Surgery initiation pressure was started at 8 mmHg for low-pressure group, and 12 mmHg for standard- pressure group, when requested by the surgeon, the pressure was raised by two points each time. Trocars were inserted on the pressure assigned by each group.

Method of pneumoperitoneum induction was left for the operating surgeon to decide as per trained, closed technique was done using veress needle, open technique was done with an infra-umbilical incision and insufflation of abdomen after 12 mm trocar insertion, optical technique utilized 12 mm optiview trocar. The trocars position was inserted for the four-trocar technique at 12 mm umbilical, 12 mm sub xiphisternum, 5 mm medial subcostal, and 5 mm lateral subcostal. For the three-trocar technique one 5 mm trocar was inserted subcostal instead of two. Trocars were introduced the the pressure set for each study group. Each operator adhered to steps of safe laparoscopic cholecystectomy with use of critical view of safety method.

Pain scores were assessed using the 11-point short pain scale (SPS-11) according to patient perception at 6 h, 12 h, 24 h, and 7th day post-operatively.

The following inflammatory markers were investigated: white blood cell (WBC) count (10^3 cells/mm), platelet (Plt) count (10^9/L), erythrocyte sedimentation rate (ESR) (millimetres per hour), C reactive protein (CRP) level (mg/L), albumin (Alb) level (g/dL), free serum cortisol level (nmol/L), interleukin − 6 (IL-6) level (pg/mL), interleukin − 17 (IL-17) level (pg/mL), interleukin − 1 beta (IL-1β) level (pg/mL), tumour necrosis factor alpha (TNF-α) level (pg/mL). Blood samples were withdrawn at the morning of surgery as baseline for all inflammatory markers. Four hours post-op cortisol levels were reassessed. Twenty-four hours post-op the remining markers were reassessed.

### Outcomes

The primary outcome of the trial was to look for a significant difference in pain on the 11-point short pain scale (SPS-11) at four set points 6-hour, 12-hour, 24-hour, and 7-days post-op. Also, to look for a significant change in the collected inflammatory markers from baseline, with a *p*-value set at 0.05. The secondary outcomes were to look for difference in surgery difficulty and operation time between the two groups.

### Randomisation, blinding and data flow

A convenient sample of 100 participants was decided, divided into 50 for each study arm. The two study arms were colour coded. The colour codes were used for randomization, data entry and analysis. All the participants, investigators, operating surgeons, observers, and analyst were blinded to the colour code reference. After closure of the study, the colour codes were disclosed to the public. Allocation was random according to a pre-set computer-generated list. Patients found to have intra operative adhesions and evidence of previous inflammation were kept in the study, ensuring no previously documented diagnosis of acute cholecystitis.

The principal investigator ensured adherence to protocol and data validity. Records were reviewed monthly, and a progress summary written and provided to study regulatory every three months. Some patients where administered dexamethasone intravenously at time of surgery induction by anaesthesia team, so subgroup analysis for dexamethasone was done to eliminate its effect on the study results.

### Statistical analysis

Analysis was conducted using SPSS version 1.0.0.1406. Gender, smoking status, ASA score, and difficulty of surgery were analyzed using Chi-square cross table. Mean duration of total operative time, insufflation time, and mean hospital-stay were analyzed using Student’s 2-tailed t-test. The mean intensity of pain was analyzed using Mann-Whitney U test. Inflammatory markers (WBC, Plt, ESR, CRP, Albumin, Cortisol, IL-6, IL-17, TNF-α, IL-1β) levels were analyzed using Welch’s t-test. Subgroup analysis for the inflammatory markers was done to eliminate the effect of dexamethasone given by anesthesia team at the time of anesthesia induction, analysis was done using Welch’s t-test. All test set *p*-value was set at 0.05 to consider results significant.

## Results

A hundred patients were included in the analysis, with 50 patients in each study arm (n = 50). (Graph 1) The sample was normally distributed. Age and BMI were analyzed using Student’s t-test (Table 1). Gender, smoking status, and ASA score were analyzed using Chi-square cross table analysis (Table 2) with a *p*-value set at 0.05.

**Table 1:** Group characteristics I

	Low-pressure group Mean ± SD	Standard-pressure group Mean ± SD	*p-value
Age (years)	43.82 ± 15.57	42.78 ± 13.53	0.722
BMI (kg/m^2^)	29.33 ± 5.57	28.78 ± 5.56	0.620

No significant difference in mean between both groups in terms of age and BMI

*p-value < 0.05

**Table 2:** Group characteristics II

		Low-pressure group n (%)	Standard-pressure group n (%)	*p-value
Gender	Male	13 (26%)	12 (24%)	0.500
	Female	37 (74%)	38 (76%)	
Smoking status	Smoker	19 (38%)	19 (38%)	0.582
	Non-smoker	31 (62%)	31 (62%)	
ASA score	I	25 (50%	21 (42%)	0.547
	II	25 (50%)	29 (58%	

No significant difference in mean between both groups in terms of gender, smoking status and ASA score

*p-value < 0.05

The mean duration of total operative time, insufflation time, and mean hospital-stay were similar across groups with no significant difference. Analysis done using Student’s t-test (Table 3) with a *p*-value of 0.05.

**Table 3:** Operation observations

	Low-pressure group Mean ± SD	Standard-pressure group Mean ± SD	*p-value
Total operative time (min)	55.0 ± 17.43	54.2 ± 19.67	0.830
Insufflation time (min)	44 ± 15.98	43.2 ± 18.20	0.807
Post-op hospital-stay (day)	1.08 ± 0.34	1.02 ± 0.14	0.253

No significant difference in mean between both groups in terms of total operative time, insufflation time and post-op hospital stay

*p-value < 0.05

None of the groups had major intraoperative complications. Three patients in the low-pressure group had a change of pneumoperitoneum initial set of pressure: two patients due to operating surgeon inexperience (senior resident) and one due to uncommon anatomy of the callout’s triangle. None had conversion to open cholecystectomy, none had more than minor intra-op blood loss, and none of the patients died during the study period. Four patients were readmitted within the 30-day period post-op: one patient from the standard-pressure group had severe vomiting and electrolyte disturbance that required in-hospital fluid resuscitation, two patients from the low-pressure group developed obstructive jaundice secondary to common bile duct (CBD) stones that required endoscopic intervention, another patient from the low-pressure group developed small bowel obstruction secondary to trocar site evisceration which was treated with reoperation and laparoscopic repair.

There was no significant difference in difficulty of surgery between low-pressure and standard-pressure groups, with a *p*-value of 0.2 obtained using Chi-square cross table (Table 4). As the trial was conducted in a teaching hospital, surgeries were done by three different types of operating surgeons: consultants, surgery fellows, and senior residents. The settings of operation were not adjusted for the study, and surgeon level of experience was chosen randomly. It was observed that no significant correlation between operator level and surgery difficulty (*p*-value = 0.369) using spearmen correlation, Spearman’s correlation (-0.091) for all cases, Spearman’s correlation (-0.221) for low-pressure group, Spearman’s correlation (-0.039) for Standard-pressure group.

**Table 4:** Difficulty of surgery and operator level of experience

		Low-pressure group n (%)	Standard-pressure group n (%)	*p-value
Difficulty of surgery	Easy	33 (66%)	38 (76%)	0.371
	Moderate	15 (30%)	8 (16%)	
	Difficult	2 (4%)	4 (8%)	
Operator level of experience	Consultant	18 (36%)	13 (26%)	0.352
	Fellow	1 (2%)	2 (4%)	
	Resident	31 (62%)	35 (70%)	

No significant difference between both groups in terms of surgery difficulty and operator level of experience

*p-value < 0.05

**Fig. 1:**
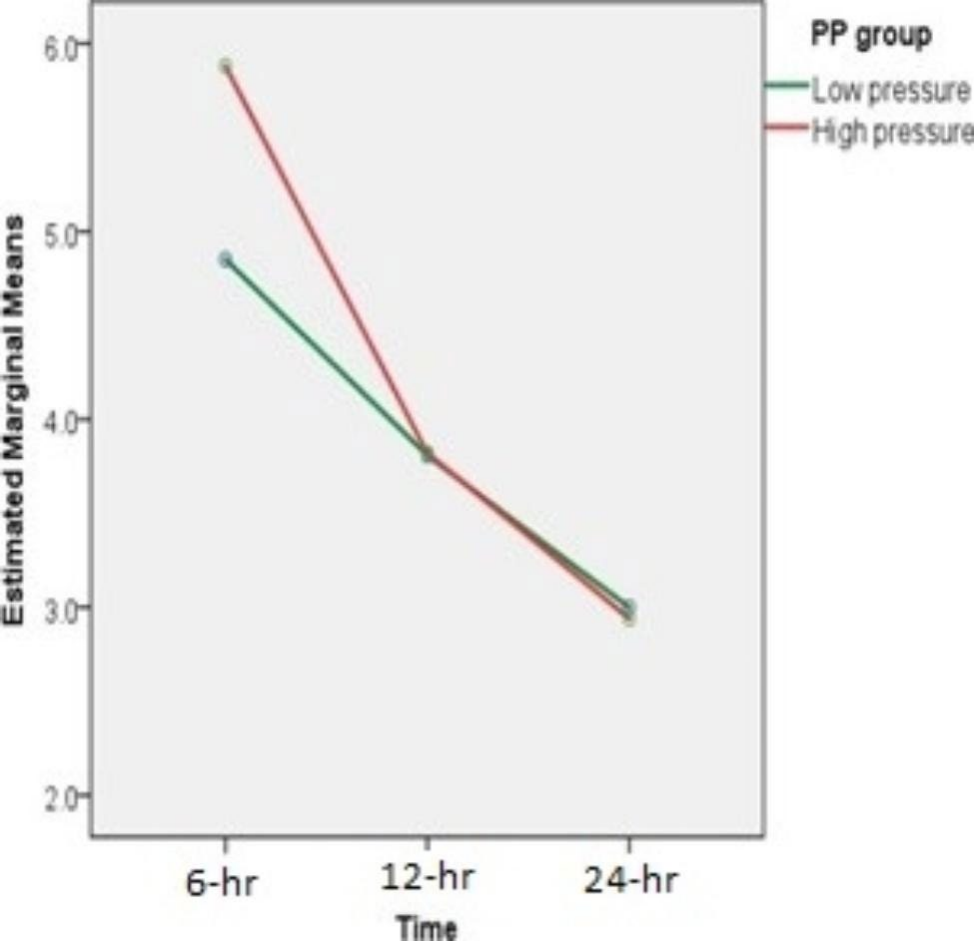
Line graph of post-operative pain score [Red line represents standard-pressure group, green line represents low-pressure group. A difference is observed at the 6-hour time point, but the mean of pain score at 12-hour and 24-hour time points is equal]

**Fig. 2:**
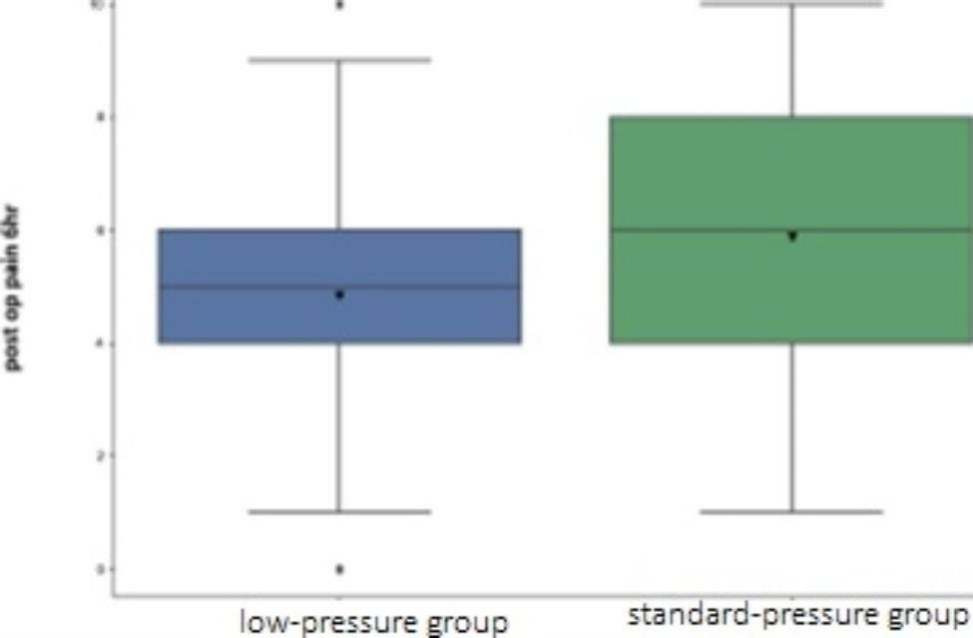
Box plot of post-operative pain at 6-houre [showing box plot of pain score at 6-hour time point, the horizontal line represents median score which is different between the two groups]

The mean intensity of pain was lower and showed significant difference at the 6-hour post-op time point in the low-pressure group with a *p*-value of 0.021 obtained using Mann-Whitney U test. (Table 5) (Figs. 1 and 2) Total analgesia count, which includes the number of times patient was given any form of analgesia either oral or intravenous post recovery, was observed and showed no significant difference across groups (*p*-value = 0.412) but the median count was lower in the low-pressure group.

**Table 5:** Post-operative pain score

		Low-pressure group (Median pain score)	Standard-pressure group (Median pain score)	*p-value
Post-op timing	6-hour	5	6	*0.021
	12-hour	3	4	0.441
	24-hour	2	3	0.273
	7th -day	0	1	0.294

A significant difference was observed between the two groups in pain score collected at 6-hour post-op time point with a *p*-value of 0.021 < 0.05, no significant difference was observed in the other time points (*p*-value > 0.05)

*p-value < 0.05

**Fig. 3:**
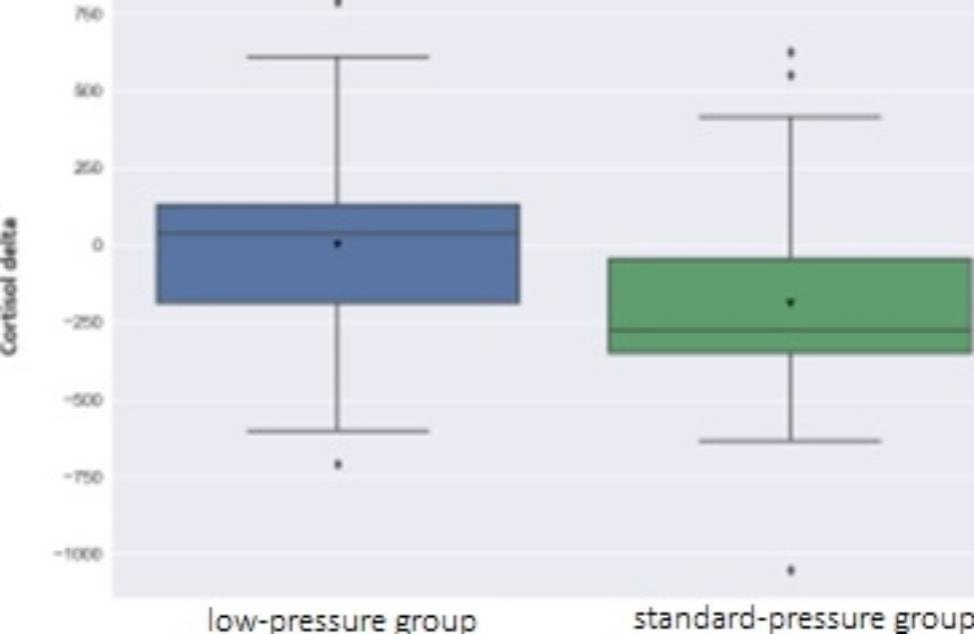
Box plot of mean difference in free serum cortisol level [Box plot shows difference in the median of change in free cortisol levels between the two groups standard Vs low pressure group, with significant difference]

Inflammatory markers were assessed pre and post operatively for both groups using Welch’s t-test and a *p*-value set at 0.05 (Table 6). A significant difference was observed in free serum cortisol levels with a *p*-value of 0.003, favoring a significant reduction of cortisol in standard pressure group with a value of 189.7 ± 306.9 (mean ± SD), compared to a value of 1.064 ± 309.64 (mean ± SD) in the low-pressure group. (Fig. 3)

**Table 6:** Inflammatory markers levels

Marker	Timing	Low-pressure group		Standard-pressure group		*p*-value
		Count	Mean ± SD	Count	Mean ± SD	
WBC (10^3^ cells/mm)	Pre op	48	7.52 ± 2.4	47	7.85 ± 2.26	0.49
	Post op	48	10.0 ± 4.1	47	10.39 ± 2.9	0.62
	∆ WBC	48	2.49 ± 3.64	47	2.53 ± 2.37	0.96
Platelet (10^9^/L)	Pre op	48	284.167 ± 6 9.94	47	275.91 ± 77.41	0.58
	Post op	48	271.188 ± 88.38	47	269.94 ± 81.65	0.94
	∆ Plt	48	-12.979 ± 53.48	47	-5.98 ± 35.63	0.45
ESR (mm/ hr)	Pre op	43	16.54 ± 12.1	43	18.98 ± 18.1	0.46
	Post op	42	20.66 ± 13.74	43	22.93 ± 16.36	0.49
	∆ ESR	43	3.65 ± 7.38	43	3.95 ± 8.65	0.86
CRP (mg/L)	Pre op	47	4.5 ± 5.51	47	6.41 ± 8.135	0.18
	Post op	47	18.85 ± 15.57	47	23.1 ± 39.45	0.49
	∆ CRP	47	14.355 ± 13.84	47	16.68 ± 38.08	0.69
Albumin (g/dL)	Pre op	47	4.42 ± 0.333	48	4.512 ± 0.28	0.144
	Post op	47	4.16 ± 0.34	48	4.25 ± 0.31	0.186
	∆ Alb	47	-0.26 ± 3.461	48	-0.265 ± 0.257	0.934
Cortisol (nmol/L)	Pre op	50	380.26 ± 228.47	49	496.17 ± 236.59	*0.015
	Post op	50	381.3 ± 272.73	49	306.48 ± 242.51	0.153
	∆ cortisol	50	1.064 ± 309.64	49	-189.7 ± 306.9	*0.003
IL-6 (pg/mL)	Pre op	45	27.82 ± 121.944	32	2.82 ± 9.643	0.25
	Post op	45	2.92 ± 4.05	32	4.89 ± 10.62	0.26
	∆ IL-6	45	-24.892 ± 121.5	32	2.079 ± 5.693	0.214
IL-17 (pg/mL)	Pre op	45	27.977 ± 42.83	32	18.735 ± 25.290	0.28
	Post op	45	28.77 ± 44.686	32	17.93 ± 25.219	0.22
	∆ IL-17	45	0.794 ± 5.253	32	-0.804 ± 5.273	0.193
IL1-β (pg/mL)	Pre op	45	50.344 ± 131.901	32	41.73 ± 84.911	0.74
	Post op	45	20.2622 ± 54.99	32	31.42 ± 48.203	0.359
	∆ IL-1 β	45	-30.082 ± 134.3	33	-9.99 ± 91.968	0.461
TNF-α (pg/mL)	Pre op	45	84.36 ± 89.982	32	89.305 ± 107.856	0.827
	Post op	45	50.44 ± 63.089	32	47.099 ± 45.122	0.798
	∆ TNF-α	45	-33.916 ± 99.85	33	-40.926 ± 103.05	0.763

Highlighted significant difference observed in level of inflammatory marker

*p-value < 0.05

Subgroup analysis was done to eliminate the effect of dexamethasone administered by anesthesia team at the time of anesthesia induction. Patients were classified into two groups: those that were given dexamethasone and those who were not (Graph 1). Analysis done using Welch’s t-test. The results showed persistence of significant difference in the free serum cortisol level in favor of standard pressure group. (Tables 7 and 8)

**Table 7:** Inflammatory markers levels, subgroup analysis for dexamethasone

Marker	Timing	Low-pressure group		Standard- pressure group		
		no Dexamethasone group				
		Count	Mean ± SD	Count	Mean ± SD	*p-value*
WBC (10^3^ cells/mm)	Pre op	22	8.10 ± 2.63	19	8.10 ± 1.66	1.00
	Post op	22	10.94 ± 3.529	19	10.18 ± 2.84	0.45
	∆ WBC	22	2.845 ± 3.824	19	2.082 ± 2.094	0.44
Platelet (10^9^/L)	Pre op	22	297.909 ± 59.063	19	278 ± 61.24	0.297
	Post op	22	277.86 ± 65.44	19	265.421 ± 65.47	0.55
	∆ Plt	22	-20.045 ± 46.781	19	-12.579 ± 29.77	0.553
ESR (mm/ hr)	Pre op	21	17.00 ± 12.87	17	21.94 ± 16.854	0.308
	Post op	20	20.15 ± 13.27	17	24.82 ± 12.177	0.275
	∆ ESR	21	2.190 ± 8.45	17	2.88 ± 8.49	0.804
CRP (mg/L)	Pre op	21	4.27 ± 5.57	19	6.06 ± 5.04	0.296
	Post op	21	17.48 ± 13.23	19	24.416 ± 29.450	0.335
	∆ CRP	21	13.209 ± 13.524	19	18.352 ± 27.200	0.447
Albumin (g/dL)	Pre op	21	4.353 ± 0.357	19	4.405 ± 0.243	0.598
	Post op	21	4.07 ± 0.3422	19	4.154 ± 0.305	0.416
	∆ Alb	21	-0.283 ± 0.2806	19	-0.251 ± 0.243	0.697
Cortisol (nmol/L)	Pre op	23	366.47 ± 258.39	20	418.459 ± 177.04	0.453
	Post op	23	339.822 ± 243.71	20	369.498 ± 249.383	0.69
	∆ cortisol	23	-26.651 ± 238.62	20	-48.96 ± 314.793	0.793
IL-6 (pg/mL)	Pre op	19	2.32 ± 6.780	13	4.845 ± 14.605	0.515
	Post op	19	3.246 ± 5.155	13	7.69 ± 15.245	0.245
	∆ IL-6	19	0.9178 ± 9.182	13	2.852 ± 6.596	0.520
IL-17 (pg/mL)	Pre op	19	38.00 ± 55.22	13	30.736 ± 24.985	0.661
	Post op	19	39.563 ± 58.054	13	28.856 ± 25.355	0.538
	∆ IL-17	19	1.555 ± 7.557	13	-1.88 ± 7.951	0.226
IL-1β (pg/mL)	Pre op	19	34.272 ± 49.926	13	28.8215 ± 29.233	0.726
	Post op	19	5.863 ± 10.926	13	21.04 ± 33.632	0.075
	∆ IL-1β	19	-28.40 ± 51.707	13	-7.781 ± 45.879	0.256
TNF-α (pg/mL)	Pre op	19	81.231 ± 78.524	13	70.49 ± 45.222	0.66
	Post op	19	41.5684 ± 34.556	13	45.971 ± 57.77	0.789
	∆TNF-α	19	-39.66 ± 70.3148	13	-24.520 ± 74.122	0.563

Showing difference in inflammatory markers for patients not given dexamethasone injection at induction of surgery. No significant difference was observed across groups

*p-value < 0.05

**Table 8:** Inflammatory markers levels, subgroup analysis for dexamethasone

Marker	Timing	Low-pressure group		Standard-pressure group		
		Dexamethasone group				
		Count	Mean ± SD	Count	Mean ± SD	*p-value*
WBC (10^3^ cells/mm)	Pre op	26	7.0412 ± 2.069	28	7.69 ± 2.610	0.317
	Post op	26	9.247 ± 4.5	28	10.526 ± 2.976	0.22
	∆ WBC	26	2.206 ± 3.543	28	2.834 ± 2.53	0.455
Platelet (10^9^/L)	Pre op	26	272.538 ± 77.206	28	274.5 ± 87.775	0.931
	Post op	26	265.538 ± 104.95	28	273.00 ± 92.06	0.782
	∆ Plt	26	-7.00 ± 85.80	28	-1.500 ± 38.99	0.685
ESR (mm/ hr)	Pre op	22	16.09 ± 11.592	26	17.04 ± 19.055	0.840
	Post op	22	21.14 ± 14.44	26	21.69 ± 18.725	0.910
	∆ ESR	22	5.045 ± 6.051	26	4.654 ± 8.845	0.861
CRP (mg/L)	Pre op	26	4.681 ± 5.577	28	6.66 ± 9.780	0.371
	Post op	26	19.96 ± 17.40	28	22.2 ± 45.514	0.815
	∆ CRP	26	15.280 ± 14.291	28	15.54 ± 44.437	0.977
Albumin (g/dL)	Pre op	26	4.47 ± 0.31	29	4.582 ± 0.285	0.176
	Post op	26	4.231 ± 0.32	29	4.31 ± 0.297	0.364
	∆ Alb	26	-0.241 ± 0.396	29	-0.269 ± 0.27	0.711
Cortisol (nmol/L)	Pre op	27	392.01 ± 203.88	29	549.77 ± 259.61	*0.015
	Post op	27	416.68 ± 295.131	29	263.023 ± 232.00	*0.034
	∆ cortisol	27	24.674 ± 362.285	29	-286.74 ± 265.36	*0.001
IL-6 (pg/mL)	Pre op	26	46.44 ± 159.00	19	1.43 ± 3.60	0.225
	Post op	26	2.69 ± 3.11	19	2.98 ± 5.44	0.220
	∆ IL-6	26	-43.75 ± 158.211	19	1.551 ± 5.10	0.520
IL-17 (pg/mL)	Pre op	26	20.65 ± 30.01	19	10.52 ± 22.59	0.223
	Post op	26	20.88 ± 30.57	19	10.46 ± 22.84	0.217
	∆ IL-17	26	0.238 ± 2.584	19	-0.067 ± 2.081	0.674
IL-1β (pg/mL)	Pre op	26	62.09 ± 168.78	19	50.56 ± 107.91	0.795
	Post op	26	30.78 ± 70.46	19	38.53 ± 55.82	0.694
	∆ IL-1β	26	-31.304 ± 172.62	19	-11.428 ± 113.624	0.641
TNF-α (pg/mL)	Pre op	26	86.64 ± 98.98	19	102.177 ± 135.06	0.658
	Post op	26	56.93 ± 77.7	19	47.87 ± 35.77	0.639
	∆ TNF-α	26	-29.717 ± 118.08	19	-51.59 ± 118.796	0.538

Showing difference in inflammatory markers for patients given dexamethasone injection at induction of surgery. Significant difference in cortisol level was observed across groups

*p-value < 0.05

## Discussion

Until today, there is no consensus on the standard pressure to operate during laparoscopic surgeries, but majority of surgical societies agrees on “the lowest intra-abdominal pressure allowing adequate exposure of the operative field rather than a routine pressure” to be used [9]. Low-pressure pneumoperitoneum, defined in literature as pressure of 10 mmHg or less, has been used to avoid the adverse physiological effects of intra-abdominal gas insufflation [10, 11].

This trial has been designed to assess laparoscopic cholecystectomy operated at a low-pressure pneumoperitoneum with a set pressure between 8 and 10 mmHg compared to a standard of 12–14 mmHg. The study design added to its strength, validity and generalisability being a prospective, randomised, double-blinded controlled clinical trial. But the study was limited to patients with low-anaesthesia risk of ASA score I and II and non-complicated gallbladder disease.

As the study was conducted at a teaching hospital, the difficulty of surgery could be assessed when operated by non-experienced surgeons in contrary to other trials that required low-pressure pneumoperitoneum surgeries to be operated by experienced surgeons. In our study sample, majority of the operations was done by non-experienced surgeons (senior residents), the distribution of surgeries done by residents was non-significantly different between the two groups. We found no correlation between the surgery difficulty and level of operator experience, indicating that low-pressure pneumoperitoneum surgery is feasible in the hands of non-experienced surgeons. Regarding the safety of low-pressure pneumoperitoneum, Mandal et al. found no significant increase in terms of difficulty of surgery, conversion rate, or post-op complication rate of low-pressure pneumoperitoneum when done in the hands of experienced surgeon. They found that the operative visual field was also not compromised [12]. Hua et al. had similar results [10]. Our trial supports similar results even in the hands of non-experienced surgeons. Mahajan et al. suggested to standardize low-pressure laparoscopic cholecystectomy in day care surgery, as they found the procedure to be safe and feasible, with no significant difference when compared to standard pressure pneumoperitoneum in terms of adequacy of surgical field, contact of parietal peritoneum to the underlying viscera during secondary port insertion, duration of surgery, complication rates, and surgery difficulty [13].

In this trial the total operative time, insufflation time and total hospital stay was similar across both groups. None of the patients had major intraoperative complication, 30-day mortality rate was zero in both groups, only 3 patients in the low-pressure group had a change from initial set pressure point, and 4 patients required re-admission within the 30-day period post-op, none of the reasons for readmission were significant nor resulted a long-term morbidity.

The benefits of operating at a low-pressure pneumoperitoneum are many and reported in multiple clinical trials 11. Post operative pain is thoroughly studied, many reported a significant reduction of post-op pain and shoulder tip pain post laparoscopic cholecystectomy among patients operated at a low-pressure pneumoperitoneum 10, 14–16. Although, a recent study by Chang et al. reported contradicting results of no significant difference in postoperative pain when operating at various pneumoperitoneum pressures, including low and very low pressures. [17] Moro et al. reported similar results. [18] Our results on pain supports the added benefit of low-pressure pneumoperitoneum of decreased pain perception post-op, our four chosen pain assessment points showed lower median pain score in the low pressure group, with a significant difference observed at the 6-hour post-op point. The total analgesia count administered post-op was lower in the low-pressure group, but we could not find a significant difference. Some other clinical trials also showed a significant reduction of post operative nausea, early resumption of oral feeding and hospital stay in low-pressure groups. ^1012^

In observing the hemodynamic effects of reduced pressure pneumoperitoneum, the heart rate, mean arterial pressure and end tidal carbon dioxide is found to have variation intraoperatively. [4] There were contradicting results of decreased vs. no impact of low-pressure pneumoperitoneum on those parameters. Dr. Vidyanand Tripathi et al. found an increase of heart rate in standard-pressure group after 10 min of surgery that was demonstrated in the low-pressure group, but this difference is not statistically significant. [19] Singh et al. demonstrated a significant difference after 10 min of surgery, and a significant difference in systolic and diastolic blood pressure after 30 min of surgery. [20] However, a study by Kanwer et al. did not find any statistical difference in systolic or diastolic blood pressure. [21] The study of hemodynamic effects of low-pressure pneumoperitoneum was out of scope and not reported in this trial.

The use of low-pressure pneumoperitoneum also demonstrated a reduced effect on derangement of hepatic blood flow 4, and significant reduction in the rise of liver enzymes. [22] Neogi et al. could observe a rise of bilirubin and liver enzymes 24 h post-op in standard-pressure pneumoperitoneum that is not demonstrated in low-pressure group, 23] recommending the use of low-pressure pneumoperitoneum in patients with impaired hepatic function. Our clinical trial did not study such effects.

The effect on immune response has been reported in few studies only, our trial was focused on ten inflammatory mediators affected by the stress of surgery. Schietroma et al. observed a significant decrease in interleukin IL-1, IL-6, and CRP. [24] Basgul et al. observed a significant lower increase in IL-6 up to 24 h after surgery, yet higher levels of IL-2 during low-pressure pneumoperitoneum. [25] In the contrary, some other trials failed to detect a benefit. [26, 27] We in this trial could detect a reduction of 8 out of 10 inflammatory markers in the low-pressure group, but the difference was not significant. Regarding cortisol hormone levels, a significant difference in its levels was present pre-operatively, this difference persisted post-op and was in favour of standard-pressure pneumoperitoneum group, this contradicting result from what is expected could be due to our poor understanding of cortisol as a stress hormone 28] and the effect of diurnal variation on the interpretation of its levels. [29] We failed to report if the surgery was done in morning or evening, as so a subgroup analysis to eliminate the diurnal variation could not be done. The levels of cortisol were assessed at morning of surgery day for all patients, but the post-op levels were assessed at a 4-hour point after the end of surgery defined as deflation of the abdomen. The rational of choosing this point is based on the known rise of cortisol as early as 4–6 h post major surgeries [30, 31].

This trial was limited to laparoscopic cholecystectomy, but in reviewing the literature we found that the beneficial effects of low-pressure pneumoperitoneum have been exploited to other surgeries. The PAROS and RECOVERY 32] trials are two trials that were made to study the effects of low-pressure pneumoperitoneum in laparoscopic colectomy in regards to pain, hospital stay, and quality of recovery. Both trials were in favour of low-pressure pneumoperitoneum usage in improving recovery after colorectal surgeries. Also, in laparoscopic pelvic surgeries it was shown that various pneumoperitoneum pressures affect the level of ovarian hormones, and this effect was observed during the first menstrual cycle post-surgery and vanishes as going towards the third menstrual cycle. [33] In laparoscopic nephrectomy, Dita Aditianingsih et al. demonstrated an attenuation of the inflammatory response, including IL- 6, in the low-pressure pneumoperitoneum group (8 mmHg) compared to standard-pressure (12 mmHg). [34].

In conclusion, this clinical trial demonstrated a reduction of pain and inflammatory markers post laparoscopic cholecystectomy in patients operated at a low-pressure (8–10 mmHg) compared to a standard-pressure (12–14 mmHg), although the difference was not significant except for the 6-hour post-op pain scores. No significant difference could be observed between the two groups regarding total surgery time, also the difficulty of surgery was not significant even in the hands of non-experienced surgeons. Thereby we recommend a larger trial to confirm the benefits of low-pressure pneumoperitoneum to establish a new standard of care in laparoscopic cholecystectomy and other similar procedures utilising intra-abdominal insufflation, and to extend the benefits to high anaesthesia risk patients such as elderly.

### Limitations

This study was conducted during the COVID pandemic, none of the patients were diseased. So the study was conducted in a small sample size due to the restrictions put on elective surgeries and limited to low-risk anaesthesia patients.

## Data Availability

The datasets used and/or analysed during the current study are available from the corresponding author on reasonable request.

## List of abbreviations

mmHg: Millimetre mercury
IRB: Institutional review board
ASA: American Society of Anaesthesiologists
SPS-11: 11-point short pain scale
WBC: White blood cell
Plt: Platelet
ESR: Erythrocyte sedimentation rate
CRP: C reactive protein
Alb: Albumin
IL-6: Interleukin − 6
IL-17: Interleukin − 17
IL-1β: Interleukin − 1 beta
TNF-α: Tumour necrosis factor alpha
post-op: Post operative
CBD: Common bile duct
COVID: COronaVIrus Disease of 2019
